# On estimating local long-term climate trends

**DOI:** 10.1098/rsta.2012.0287

**Published:** 2013-05-28

**Authors:** S. C. Chapman, D. A. Stainforth, N. W. Watkins

**Affiliations:** 1 Department of Physics, University of Warwick, Coventry, UK; 2 Grantham Institute, London School of Economics, London, UK; 3 British Antarctic Survey, Cambridge, UK; 4 Centre for the Analysis of Timeseries, London School of Economics, London, UK

**Keywords:** climate change, local climate sensitivity, uncertainty, prediction, impacts

## Abstract

Climate sensitivity is commonly taken to refer to the equilibrium change in the annual mean global surface temperature following a doubling of the atmospheric carbon dioxide concentration. Evaluating this variable remains of significant scientific interest, but its global nature makes it largely irrelevant to many areas of climate science, such as impact assessments, and also to policy in terms of vulnerability assessments and adaptation planning. Here, we focus on local changes and on the way observational data can be analysed to inform us about how local climate has changed since the middle of the nineteenth century. Taking the perspective of climate as a constantly changing distribution, we evaluate the relative changes between different quantiles of such distributions and between different geographical locations for the same quantiles. We show how the observational data can provide guidance on trends in local climate at the specific thresholds relevant to particular impact or policy endeavours. This also quantifies the level of detail needed from climate models if they are to be used as tools to assess climate change impact. The mathematical basis is presented for two methods of extracting these local trends from the data. The two methods are compared first using surrogate data, to clarify the methods and their uncertainties, and then using observational surface temperature time series from four locations across Europe.

## 1. Introduction

That increases in atmospheric greenhouse gases will lead to global warming and climate disruption at a level that poses a threat to society is clear. The amount of warming to expect on a global scale during the twenty-first century under any particular concentration scenario is, however, uncertain. It depends partly on climate sensitivity that is defined as the equilibrium change in the annual mean global surface temperature following a sustained doubling of the atmospheric carbon dioxide concentration [1]. For any given change in global mean temperature, a wide variety of changes are possible at local scales. Yet it is at local scales that the impacts of climate change will be felt directly and at which adaptation planning decisions must be made [2]. Many planners and environmental disciplines are aware that an assumption of stationarity is ill-founded; therefore, basing decisions on climatic distributions over the last century is likely to be sub-optimal for the climate of today and even more so for the climate of the future [3]. The question for planners is how to obtain better information to support their assessments and decisions. The simulations of complicated global climate models can suffer from serious questions of robustness and reliability [4–6], and are known to inadequately represent behaviour that will have an impact on local scales. They are, therefore, a questionable basis for decision-making [2,4,7]. Our aim herein is to present a method for analysing local climatic time-series data to assess which quantiles of the local climatic distribution show the greatest and most robust trends over the last 60 years. A local trend parameter is constructed to automatically incorporate all the effects of wider regional and global changes while allowing for the local geographical factors that affect the local climate.

The local trend parameter proposed here isolates the change that has occurred in a single set of at-a-point observations of a given climate variable, for example, maximum daily temperature, over the available observation period (here, 60 years). The parameter that we obtain is a function of both geographical location and the given variable (or occurrence likelihood of the given variable); these are treated as independent. We thus obtain as outputs how the trend varies with temperature (or occurrence likelihood), and with geographical location. The trend parameter isolates the change in the climate variable, say temperature, at a fixed likelihood, and hence is closely related to the change in event return time. The novelty of our approach is its simplicity. It offers simple quantitative understanding of what the data can tell us about past changes and how robust they are. The trend parameter is calculated independently for each location; no geographical correlation is assumed. Thus, as outputs it identifies where trends are consistent across extended geographical regions, and which regions do not show a clear signal.

Our approach is thus distinct from, and complementary to, inference-based approaches that combine multiple observations and evidence [8] or combine spatio-temporal trends ([9]; see also [10]). Importantly, it has relevance for the recent debate on the *perception* of how the climate has changed [11] in that it quantifies how changes in climate have played out on local scales and what the uncertainties are, simply from a given set of observations, independent of models or other inferences. As our method is model independent and relies entirely on the data, it does not distinguish between the many components that can influence local climate. When combined with understanding of climatic trends expected for the future, where such trends can be identified and understood, this local measure can assist in prioritizing adaptation projects and making judgements over the relative risks of different options.

This quantile-dependent change may or may not be representable by a change in the mean and/or a few higher moments of the distribution. Given the nonlinear nature of the system, we might expect that it would not. Furthermore, variations in the distribution can occur on a wide range of time scales. The mathematical challenge is, therefore, to make best use of the data to quantify the changes, to identify the robust aspects of the results and when the change cannot be well quantified. This paper addresses these challenges. In §2, we derive the local trend parameter and present two methods for extracting it from time-series data. In §3, the methods are illustrated using surrogate climate data designed to illustrate the concepts and the challenges of extracting a clear signal given statistical variations. Section 4 illustrates its application to real data and gives methods for quantifying robustness. The conclusions present opportunities for further development and application of the technique. The relationship to, and representation of, the local long-term trend in terms of return times is covered in appendix A.

## 2. Method: a parameter for local long-term trends

Our starting point is that we have a daily observation of some variable, say mean or maximum or minimum temperature, *T* at different geographical locations and over an extended interval of time. There is a climatic distribution from which these observations are drawn. This local distribution will vary with time, in a manner that depends upon the changing state of the climate at all scales. We represent the climate state at any given time *t* by the function *g*(*t*).

**Figure 1.**
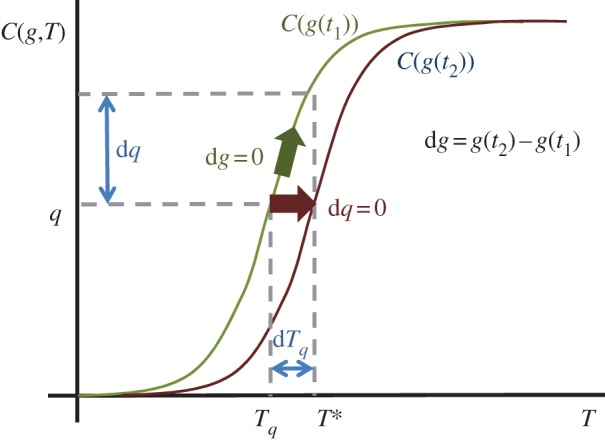
Temperature *T*_*q*_ is observed with likelihood *q*. If the temperature cdf is not changing with time (*g* is constant) then temperature T* is from the same distribution as temperature *T*_*q*_, and is observed with likelihood *q*+d*q*. This is the path d*g*=0. If the temperature cdf is changing with time, due to change in *g*(*t*), then temperature T* is observed at time *t*_2_ with the same likelihood *q* as temperature *T*_*q*_ was observed at *t*_1_. This is the path d*q*=0. (Online version in colour.)

An underlying assumption is then that the effect of changing *g* on the distribution of observations of *T* is on a much longer time scale than that over which individual samples of *T* distributions are obtained. For each location, we can aggregate daily seasonal temperature observations over some multi-year interval *τ*, centred on time *t*, to obtain a cumulative density function (cdf). Let us assume that the cdf *C*(*T*,*g*(*t*)) can be treated as a continuous function of temperature and of time-dependent climate state *g*. Then at a given time *t* with corresponding *g*(*t*), the cdf value *q* is the likelihood of the quantile *T*_*q*_ of a given temperature observation *T*≤*T*_*q*_:
2.1
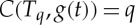
and
2.2
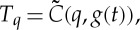
where 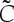 is the quantile function obtained by inverting the cdf with respect to temperature. We can write the variation, to leading order:
2.3
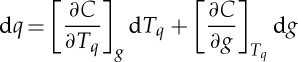
and
2.4
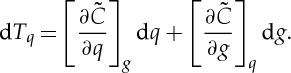
At some later time, we observe a temperature T* so that d*T*_*q*_=T*−*T*_*q*_; we will assume that this can be treated as a small change. We can in principle ask for the T* that could occur in the absence of any change in climate state, that is d*g*=0, directly from the change in the quantile function; from (2.4) this is
2.5
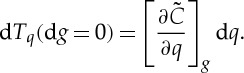
We can also ask for the contribution to the observation T* solely owing to the change in climate state, that is at d*q*=0, from the quantile function. From (2.4) this is
2.6
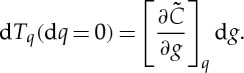
This is illustrated in figure 1. A sufficiently accurate approximation for the quantile function at two times *t*_1_ and *t*_2_ would directly give an estimate of the discrete change Δ*T*_*q*_ over an interval [*t*_1_,*t*_2_]. We now approximate d*T*_*q*_ by Δ*T*_*q*_ by using
2.7
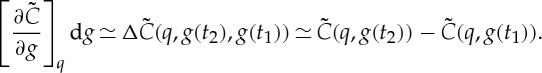
This direct estimate of the change Δ*T*_*q*_ due to the change in *g* requires full knowledge of the quantile function, that is, the inverse of the cdf. In practice, given that there is a fundamental upper limit on the number of observations in a given season, this can be problematic, particularly in the tails of the distribution for non-Gaussian processes. We can instead obtain an expression where a direct estimate of the cdf is needed. Setting d*q*=0 in (2.3), we obtain
2.8
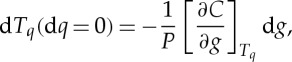
where *P*(*T*_*q*_,*g*(*t*)) is the probability density function (pdf). Now let us compare two different realizations of the cdf obtained at different times *t*_1_ and *t*_2_ over which there is the discrete change Δ*T*_*q*_. Then, we will approximate d*T*_*q*_ by Δ*T*_*q*_ using
2.9
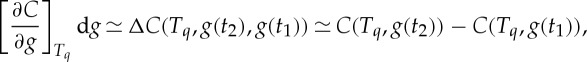
then
2.10
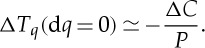
This expression suggests pragmatic strategies for estimating a *local trend parameter* *S*=Δ*T*_*q*_(d*q*=0). This parameter is a function of the observed variable, here temperature *T*, and of the geographical location at which *T* is observed. It parametrizes which parts of the distribution are changing most rapidly with change in climate state *g*. We can immediately see that the pdf acts as an amplification factor for the local trend parameter. A given change in cdf will be most impactful where the pdf is small, that is, in the tails of the distribution rather than at the mean. Importantly, in a finite time interval of data, the pdf can be estimated across an aggregate of the entire dataset, whereas the estimate of the change in the cdf necessitates smaller sized samples. In sparse datasets, such as precipitation, an estimate in the change in cdf may not be feasible. However, it may still be possible to estimate the pdf aggregated over the entire interval. The geographical variation of the pdf alone contains information on the geographical variation of the local trend parameter. Alternatively, we can write (2.10) in terms of the change in return time *R*(*T*_*q*_) of events of size *T*_*q*_ (see appendix A):
2.11
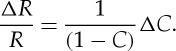

## 3. Illustration with synthetic data

We first illustrate the method, and how it can be used to optimize against observational statistical uncertainty by constructing a test time series of pseudo-daily observations that is of a similar finite size as the data as follows. We have daily observations over a ‘season’ which is typically no longer than three months, over a period of typically 50–100 years. For each of 100 ‘years’ of the test time series, we then represent the ‘season’ by a sample of 100 independent, identically distributed random numbers that are drawn from a pdf which is the Gamma distribution (where *γ* is the Gamma function):
3.1
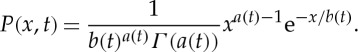
For each ‘year’ *t*, that is, each 100 element sample, we specify the shift and scale parameters *a*(*t*) and *b*(*t*), where *t*=[1,2,3,…,100]. These determine the mean (*ab*), the variance (*ab*^2^), the skewness (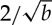) and the excess kurtosis (6/*b*). To model a distribution with slowly changing *g*(*t*), we will consider a linear change in *a* and/or *b* from one ‘year’ to the next.

**Figure 2.**
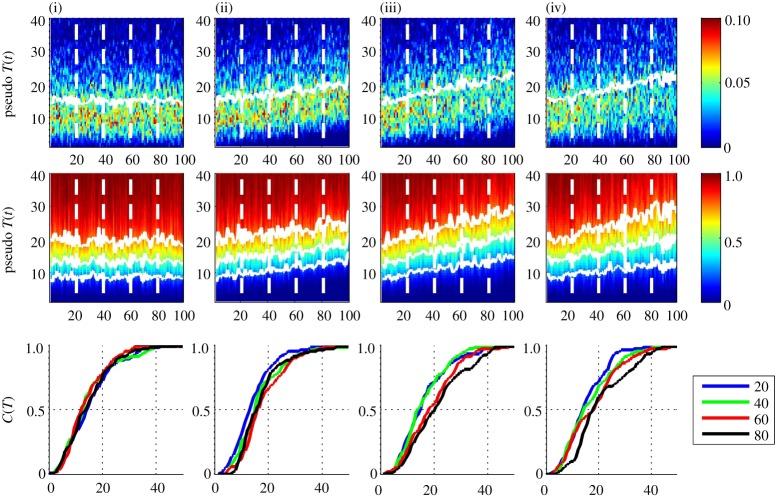
Pseudo-annual temperature data with (i) no time variation, (ii) constant shift at all temperatures, (iii) mean and variance increase with time and (iv) mean, variance, skew and kurtosis increase with time. The top panel shows the ‘yearly’ (100 sample) pdfs with temperature as ordinate and time as abscissa, with the mean indicated by a white line; the colour bar is pdf value. The middle panel shows the ‘yearly’ (100 sample) cdfs, same axes, with 0.25, 0.50 and 0.75 quantiles indicated by solid white lines; colour bar is cdf value. The four vertical dashed lines at *t*=[20,40,60,80] indicate the years for which individual cdfs are plotted in the bottom panel. Bottom panel plots these four individual cdfs with temperature as abscissa. (Online version in colour.)

Figure 2 shows 100 ‘years’ of this pseudo-temperature data. The column (i) is for 100 samples (‘years’) with the same constant *a*=*a*_0_=3 and *b*=*b*_0_=5, representing a time series with no underlying change in climate state *g*(*t*). The column (ii) is the same samples but with each shifted such that 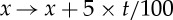; this is a uniform trend in time across the entire distribution. The column (iii) has *b* constant and *a*(*t*)=*a*_0_[1+1/2(*t*/100)], so that the mean and variance both increase with increasing years *t*, and column (iv) has *a* constant and *b*(*t*)=*b*_0_[1+1/2(*t*/100)], so that the first four moments all increase with increasing years *t*. The pseudo-temperature series in column (iv) has an approximately linear trend in these quantiles.

**Figure 3.**
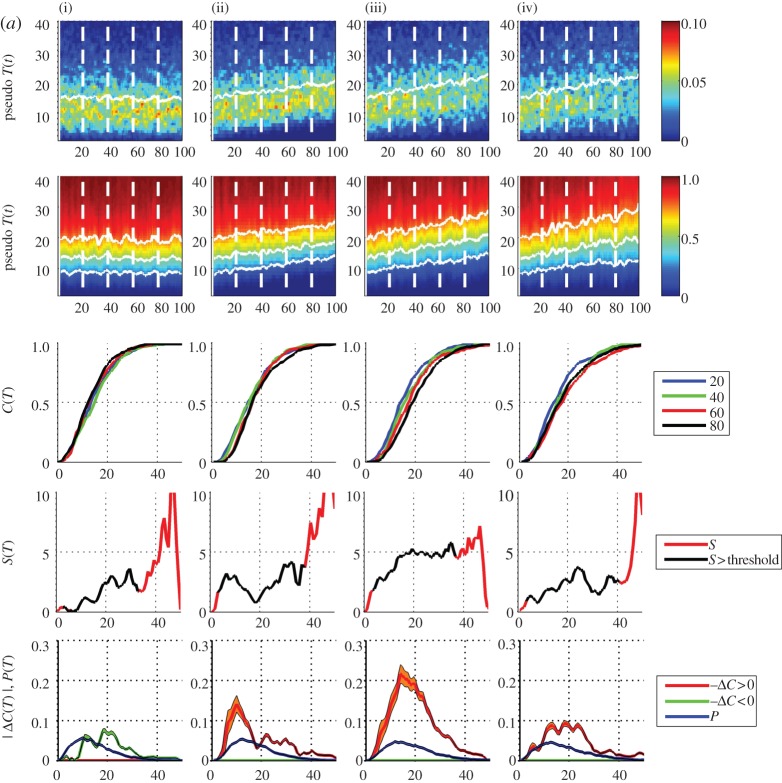

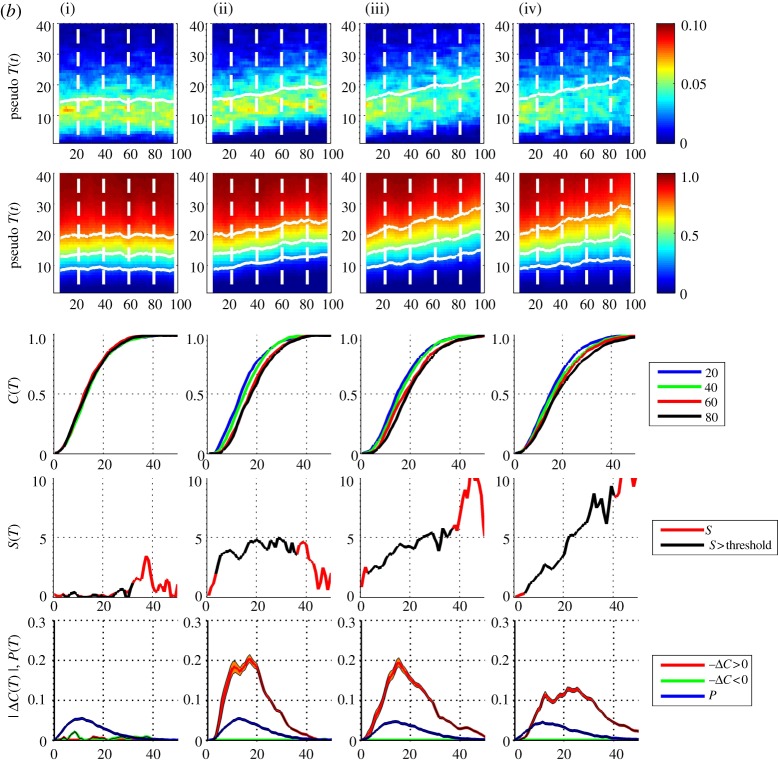
(*a*) Pseudo-annual temperature data, (i–iv) and top three rows are as figure 2 except that the pdfs and cdfs are *τ*=3 ‘year’ aggregates (300 samples). The fourth row plots the local trend parameter with temperature as abscissa (red line); this is overplotted with a black line, where both pdf and |Δ*C*| values exceed a threshold (black line). The bottom row plots the pdf from aggregate over the entire dataset (blue line) and −Δ*C* (orange line) and +Δ*C* (green line) from the 3 year aggregates; histogram uncertainties are indicated by shading, estimated as 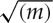 where *m* is the number of samples per pdf histogram bin. (*b*) Pseudo-annual temperature data, as per (*a*) except with *τ*=9 year aggregates. (Online version in colour.)

Clearly, there is considerable statistical variation in the pdfs, and to a lesser extent, the cdfs, as a consequence of small yearly sample size. To increase the sample size, we need to aggregate observations over several consecutive years. Our expression (2.10) allows us to optimize this separately for the pdf and the cdf. A procedure for this is shown in figure 3. The top two rows of panels show the same time series as figure 2, but now the pdfs and cdfs are of aggregates over 3 (figure 3*a*) and 9 (figure 3*b*) years, respectively, the abscissa now indicates the central year *t* of each aggregate. The set of four individual cdfs now show trends which are particularly clear in the 9 year aggregate cdfs; in our simple model for a temperature time series, we can see that a roughly linear positive trend in the mean and variance of a pdf with non-zero skew leads to the largest change in the positive upper quadrant of the cdf, rather than at the mean. If the skew and kurtosis also have a positive trend the largest change in the cdf moves further to large positive values.

The bottom rows of panels of figure 3*a*,*b* refer to the indirect estimation of the local trend parameter from expression (2.10) above. To estimate the pdf, we have aggregated over the entire set of samples, that is, the full dataset. The difference Δ*C* is taken between the cdfs estimated from samples centred on *t*=80 and *t*=20. These samples for each of the pair of cdfs are over (figure 3*a*) *τ*=3 and (figure 3*b*) *τ*=9 ‘years’, respectively.

In the fourth row of panels, the ratio *S*=−Δ*C*/*P* obtained from these is plotted (red line) for all values. Clearly, when either −Δ*C* or *P* is small, this ratio is dominated by statistical uncertainty and we have overplotted (black line) values where the magnitudes of both Δ*C* and *P* exceed a threshold value of 0.005. For arbitrarily good statistics, these plots should show the following, from left to right: column (i) 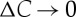, so 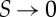, (ii) *S* constant, (iii) and to a greater extent (iv) *S* is largest for values of *x* greater than the mean as the positive skew and kurtosis both increase with *t*.

**Figure 4.**
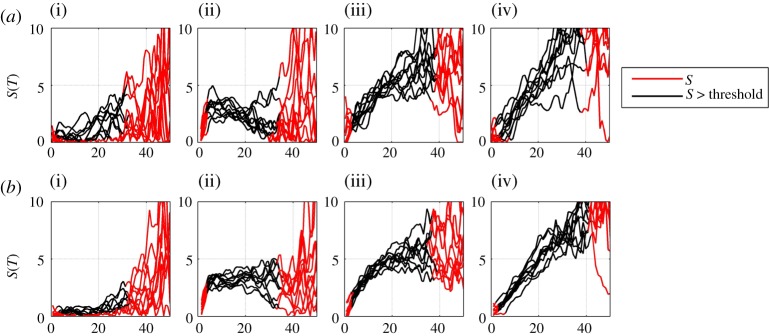
(*a*) Indirect estimate of local trend parameter for 10 realizations of pseudo-annual temperature data with 3 year aggregates used to estimate the cdf. The model data parameters (i–iv) are as in previous figures. (*b*) Indirect estimate of local trend parameter for 10 realizations of pseudo-annual temperature data with 9 year aggregates used to estimate the cdf. The model data parameters (i–iv) are as in previous figures. (Online version in colour.)

Increasing the sample interval *τ*, over which the cdfs used to obtain Δ*C* are estimated, improves statistics. This procedure is valid provided that this interval *τ* is much shorter than that over which *g*(*t*) is changing. Importantly, in real data, this procedure effectively removes trends on time scales shorter than *τ*. The choice of *τ* thus needs to be optimized for the specific dataset under consideration. We demonstrate this using the pseudo-temperature data in figure 4*a*,*b* for *τ*=3 and *τ*=9 ‘years’, respectively. In these plots we calculate the local trend parameter *S* for 10 successive realizations of the pseudo-temperature data, that is, we increment *t*_1_ and *t*_2_ by one year for each curve that is plotted, keeping the interval *t*_2_−*t*_1_ fixed. For arbitrarily large sample size these curves should collapse on top of each other. This suggests an operational measure for the reliability of the local trend parameter; one can consider a given value of the parameter to be reliable provided that value is exceeded in all (here 10) realizations. A further check on the robustness of the trend in time is to construct a surrogate dataset (see also [12]), that is, to recalculate the local trend parameter having randomly shuffled the time order in which the samples are observed; this quantifies the observed local trend parameter value that could occur randomly with these finite sample statistics. We will illustrate these methods using the E-OBS data in §4.

**Figure 5.**
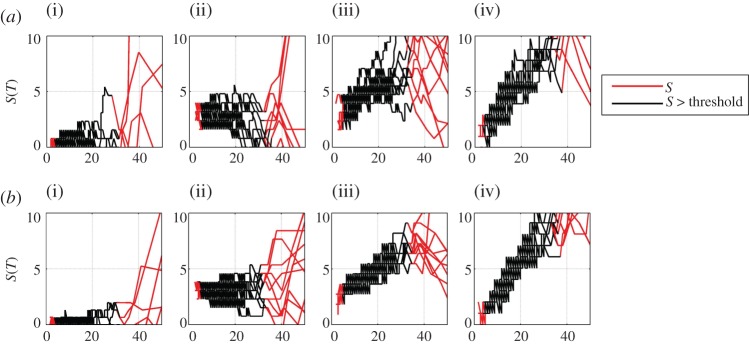
(*a*) Direct estimate of local trend parameter for 10 realizations of pseudo-annual temperature data with 3 year aggregates used to estimate the cdf. The model data parameters (i–iv) are as in previous figures. (*b*) Direct estimate of local trend parameter for 10 realizations of pseudo-annual temperature data with 9 year aggregates used to estimate the cdf. The model data parameters (i–iv) are as in previous figures. (Online version in colour.)

We finally compare this method with direct estimation of the local trend parameter using expression (2.6). This is shown in figure 5*a*,*b,* which are in the same format as figure 4*a*,*b,* but where we have numerically inverted the cdf to obtain the quantile function. We plot the difference in quantile function with all values plotted in red, and values for 0.05<*C*<0.95 plotted as a black line as an indication of the limits of validity of the method; this is not a firm quantitative estimate of uncertainty and, in practice, one would need to obtain this from an analysis of the counting statistics and other uncertainties in the data. We can see that the indirect method and direct methods correspond within errors; the discretization or ‘stepping’ in figure 5 is a direct consequence of the process of numerical inversion of the cdf.

## 4. Application: E-OBS temperature data

We now illustrate this methodology with daily temperature measurements from the E-OBS gridded dataset [13] (1950–2011 v.5.0). We will look at four locations chosen arbitrarily, but to be sufficiently far from each other to show geographical variation, at longitude and latitude (i) [4.75 52.25] Leiden, The Netherlands, (ii) [−4.75 51.75] west Wales, (iii) [−4.75 42.75] Leon, north Spain and (iv) [11.25 43.75] Florence, Italy. We will repeat the above procedure using data from these four locations, focusing on summer; aggregating daily temperatures over three month intervals within each year of observation (June–August) each year gives a sample of 92 observations per year.

**Figure 6.**
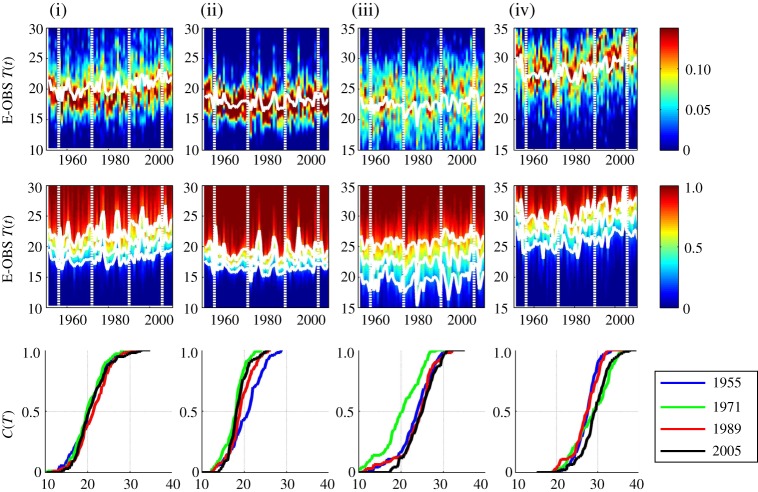
E-OBS annual temperature data at four locations (i–iv). Panels show from top to bottom: yearly summer (92 sample) pdfs with temperature as ordinate and time as abscissa and a solid white line indicating the mean; yearly (92 sample) cdfs, same axes, solid white lines indicating the 0.25, 0.5 and 0.75 quantiles; the vertical dashed white lines indicate 4 years for which four individual cdfs are plotted in the bottom panels with temperature as abscissa. (Online version in colour.)

**Figure 7.**
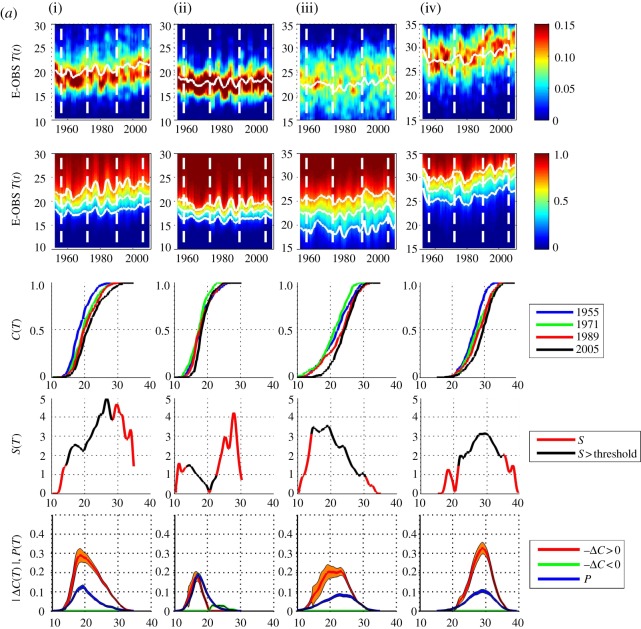

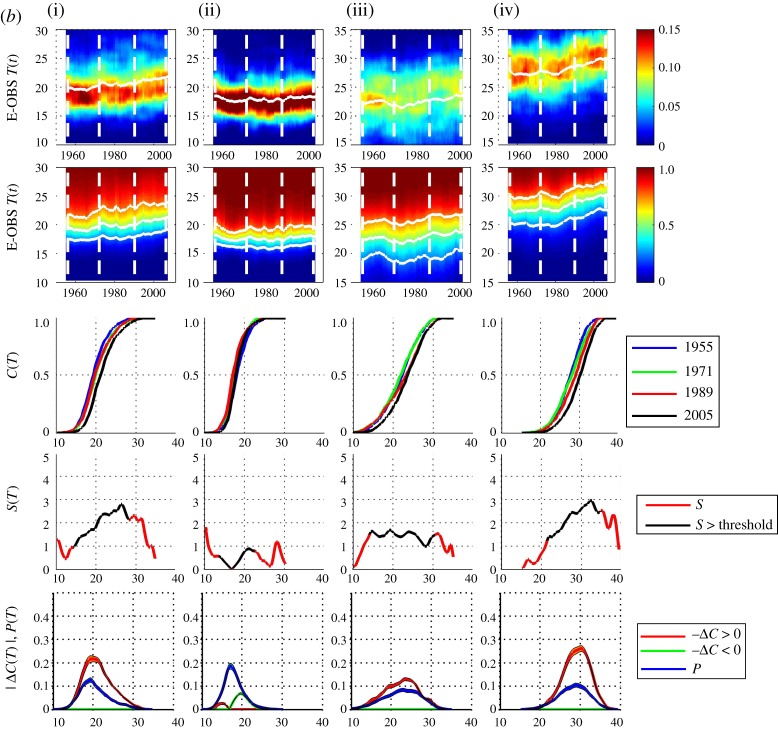
(*a*) E-OBS temperature data, for four locations (i–iv) in the same format as figure 3 with *τ*=3 year aggregates. (*b*) E-OBS temperature data, as per (*a*) except with *τ*=9 year aggregates. (Online version in colour.)

Figure 6 shows the pdfs and cdfs for these yearly samples in the same format as figure 2. Figure 7*a*,*b* are in the same format as figure 3*a*,*b* showing the data with sample *τ*=3 and 9 years, respectively. As above, a sample centred on year *t* will for *τ*=3 include years *t*−1,*t*,*t*+1 and for *τ*=9 include years *t*−4,*t*−3,…,*t*+4; so that, for *τ*=3 the sample centred on 1955 is over years 1954–1956, and for *τ*=9, over years 1951–1959 inclusive. Here, Δ*C* is then estimated using two samples centred on years 1955 and 2005. The pdf is estimated from the entire dataset in the same manner as for the test time series.

Distinct behaviour can be seen at these four locations. In figure 7*b*, The Netherlands (i) shows a clear skew towards higher temperatures and peaks at the largest temperatures where the trend is reliable (i.e. within the threshold); at the quantile ∼25^°^–28^°^ there is a positive shift of ∼2^°^–3^°^. West Wales (ii) shows no significant local trend. North Spain (iii) shows a local trend of ∼1^°^–2^°^ across all quantiles, and Florence (iv) has the same functional dependence upon quantile as The Netherlands, but at higher temperatures; at the quantile ∼27^°^–35^°^ there is a positive shift of ∼2^°^–3^°^.

**Figure 8.**
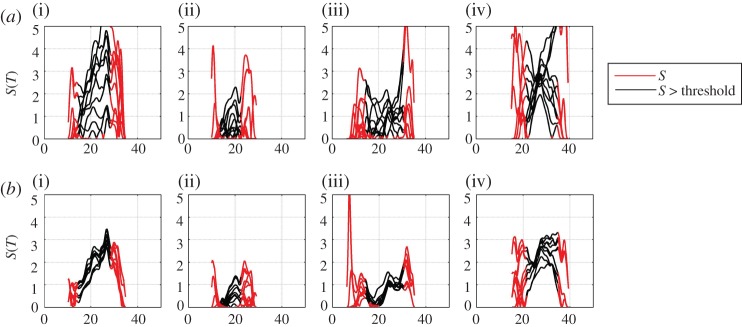
(*a*) Indirect estimate of local trend parameter for 10 realizations of E-OBS temperature data with 3 year aggregates used to estimate the cdf. The locations (i–iv) are as in previous figures. (*b*) Indirect estimate of local trend parameter for 10 realizations of E-OBS temperature data with 9 year aggregates used to estimate the cdf. The locations (i–iv) are as in previous figures. (Online version in colour.)

**Figure 9.**
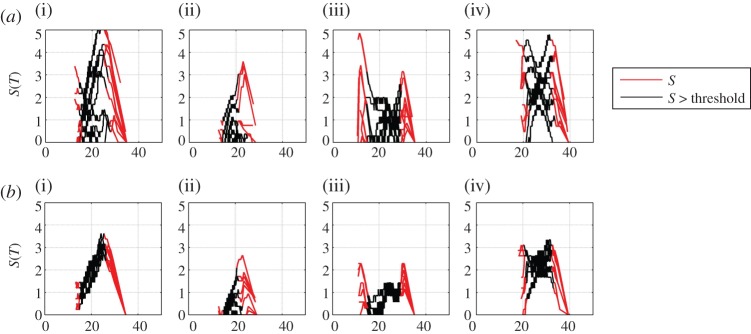
(*a*) Direct estimate of the local trend parameter for 10 realizations of E-OBS temperature data with 3 year aggregates used to estimate the cdf. The locations (i–iv) are as in previous figures. (*b*) Direct estimate of the local trend parameter for 10 realizations of E-OBS temperature data with 9 year aggregates used to estimate the cdf. The locations (i–iv) are as in previous figures. (Online version in colour.)

Figure 8*a*,*b* shows the local trend parameter estimated for 10 successive intervals in the same manner as above, for *τ*=3 and 9 years, respectively. For each interval, we take two samples centred on year *t*_1_ and *t*_2_ and we will use the same *t*_2_−*t*_1_ for all 10 intervals. The first estimate of Δ*C* is made using samples centred on *t*_1_=1955 and *t*_2_=1995, the second, on samples centred on *t*_1_=1956 and *t*_2_=1996, and so on. In figure 8*a*, we show the result of this procedure for samples with *τ*=3, so that the first estimate of Δ*C* is made using two samples aggregated over the years 1954–1956 and 1994–1996, the next estimate over 1955–1957 and 1995–1997, and so on. Figure 8*b* shows the same procedure with *τ*=9. Here, there is both variability due to the finite size of the sample as seen in the model data above, and in addition, systematic trends due to departures from our simple assumption (2.10); by taking a lowest order expansion, we have assumed that the trends can be approximated as linear. We have recalculated the curves of figure 8*a*,*b* using the direct estimate of the local trend parameter (2.6) and these are shown in figure 9*a*,*b*. Again, within uncertainties, these track the behaviour seen in figure 8*a*,*b*.

**Figure 10.**
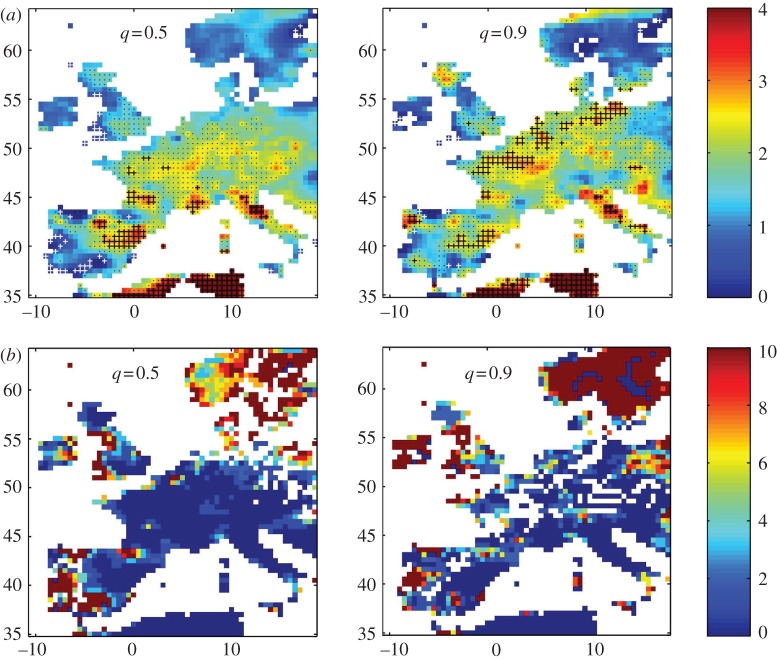
(*a*) Local trend parameter *S*(longitude,latitude,*q*) in degrees centigrade (indicated by the colour bar) at *q*=0.5 and *q*=0.9 for a subset of the E-OBS data. Robustness of these geographical trends (see text) is indicated by locations where in all 10 realizations of this map the magnitude of *S*>2 (black crosses), *S*>1 (black dots) and *S*<1 (white crosses). No data and regions where either |Δ*C*| or *P* fall below the threshold are indicated as white space. (*b*) Percentage likelihood (indicated by the colour bar) that the local trend parameter *S*(longitude, latitude, *q*) at *q*=0.5 and *q*=0.9 for a subset of the E-OBS data shown in (*a*) could occur randomly, estimated for 100 realizations of a surrogate dataset obtained by random shuffling of years (see text). No data and regions where either |Δ*C*| or *P* can fall below the threshold are indicated as white space. (Online version in colour.)

The local trend parameter (here, for temperature) provides both geographical and at a quantile variation as outputs. While the dependence on quantile is most easily seen at a given location as shown above, geographical dependence can be seen using maps. We can map the local trend parameter either at fixed quantile (temperature) or at fixed quantile function and the latter case is shown in figure 10*a*. Here, for the E-OBS data, we plot, as a colour map, the value of *S*(longitude,latitude,*q*) in degrees centigrade (indicated by the colour bar), at *q*=0.5 and *q*=0.9, with Δ*C* estimated using samples centred on years *t*_1_=1958 and *t*_2_=2001 with *τ*=9 years (so that Δ*C* is obtained from two values of *C* from samples over years 1954–1962 and 1997–2005). On these plots, locations where either |Δ*C*| or *P* fall below the threshold are indicated as white space. Clear geographical trends can be seen, which vary with quantile function. Detailed interpretation of these maps will be presented in future work; here, we focus on two methods to quantify how robust these trends are. First, we can recalculate *S*(longitude,latitude,*q*) as above, with Δ*C* estimated using 10 samples centred on years *t*_1_=1955,1956,1957,… and *t*_2_=1995,1996,1997,… with the same *t*_2_−*t*_1_. We then indicate on the maps shown in figure 10*a* locations (longitude,latitude) where in all 10 samples the magnitude of *S*>2 (black crosses), *S*>1 (black dots) and *S*<1 (white crosses). This graphically indicates robustness in the trend in Δ*C* over time. Second, we will estimate how significant the observed values of *S*(longitude,latitude,*q*) are compared with that obtained from a random variation in Δ*C* over time (a surrogate data method, see [12]). We recalculate 100 realizations of the map shown in figure 10*a* with the years of the E-OBS data randomly shuffled. We then for each (longitude,latitude,*q*) count the number of times *N*_r_ the value of *S* in the randomized realization exceeds that seen in the original data. We then map *N*_r_(longitude,latitude,*q*) in figure 10*b*. The colour map can be directly read as the likelihood (percent) that the local trend *S*(longitude,latitude,*q*) shown in figure 10*a* could occur at random. We see that throughout most of central Europe, this likelihood is below 2 percent, and generally is large where *S* is small.

## 5. Conclusions

We have presented a method which provides an improved way of understanding the observed consequences of a globally changing climate at specific locations and for specific thresholds. It provides a methodology for informing user-specific decisions that are often vulnerable to specific thresholds. It avoids the use of complicated models and all the epistemic uncertainties which that can involve. There are further interesting mathematical challenges in applying it to different types of distributions such as those representing precipitation, multi-variable distributions and functions of multiple variables. There are also, of course, opportunities to apply the method to thresholds experienced in real-world situations. These could be as diverse as building design, infrastructure vulnerability, strategic planning for heat waves, changing risks of crop failure, impacts on food prices, etc. These opportunities are intrinsically multi-disciplinary in nature and provide an exciting and rich vein of new research opportunities to support science-based policy and decision-making.

## Appendix A. Return times

The above formalism is directly related to the return time of events. In time period Δ*t*, we observe *N* events which in ascending size order are *x*_1_…*x*_*k*_…*x*_*N*_, and have a mean time between observations of *δt*=Δ*t*/*N*. Then within period Δ*t*, there are *N*−*k*+1 events of size *x*≥*x*_*k*_. The return period or return time *R* for an event ‘at least as big as *x*=*x*_*k*_’ is then (see also [14])

A1

for (*N*−*k*) large. In this large (*N*−*k*) limit, the cdf *C*(*x*=*x*_*k*_)=*k*/*N* so that

A2

Note that the return time is only sharply defined for sufficiently large (*N*−*k*); if the underlying statistics were Gaussian, then the percentage error is . Practically, it fails at the largest events .

Now, consider the slowly driven system with cdf *C*(*x*,*g*(*t*)); there will be a corresponding return time *R*(*x*,*g*(*t*)). The changes with *x* and *g* are

A3

and

A4

If we define the change in return time due to change in *g* alone, in the same manner as Δ*C* above, so that

A5

then using (A2)

A6

Since from above, the local trend parameter d*x*(d*q*=0)=−(1/*P*)(∂*C*/∂*g*)=−Δ*C*/*P*, we can express the local trend parameter in terms of the change in the return time due to change in *g*:

A7

Finally, we can check that Δ*R* is indeed the change in return time due solely to change in climate state. We explicitly denote *q*=*C*(*x*,*g*) as above, and the return time as *r*=*R*(*x*,*g*), this has variation:

A8

and we have

A9

Now, the change in return time due to change in climate state *g* only is that at d*q*=0. From (A3) and (A4), we can see that d*q*=0 is just d*r*=0; the total variation in return time is zero. However, the change in return time due to change in climate state alone is, at d*r*=0 from (A8)

A10

which using (A3) is just

A11

which at d*q*=0 is just

A12

as above.
